# In Vitro Inhibitory Effects of Synthetic Cannabinoid EAM-2201 on Cytochrome P450 and UDP-Glucuronosyltransferase Enzyme Activities in Human Liver Microsomes

**DOI:** 10.3390/molecules23040920

**Published:** 2018-04-16

**Authors:** Tae Yeon Kong, Soon-Sang Kwon, Jae Chul Cheong, Hee Seung Kim, Jin Young Kim, Hye Suk Lee

**Affiliations:** 1 BK21 PLUS Team for Creative Leader Program for Pharmacomics-Based Future Pharmacy, Drug Metabolism & Bioanalysis Laboratory, College of Pharmacy, The Catholic University of Korea, Bucheon 14462, Korea; kongtaeyun@naver.com (T.Y.K.); zuzutnseo@naver.com (S.-S.K.); 2 Forensic Chemistry Laboratory, Forensic Science Division, Supreme Prosecutor’s Office, 157 Banpo-daero, Seocho-gu, Seoul 06590, Korea; Saturn-jjc@spo.go.kr (J.C.C.); hskjay@spo.go.kr (H.S.K.); paxus@spo.go.kr (J.Y.K.)

**Keywords:** EAM-2201, drug–drug interaction, human liver microsomes, cytochrome P450, UDP-glucuronosyltransferase

## Abstract

EAM-2201, a synthetic cannabinoid, is a potent agonist of the cannabinoid receptors that is widely abused as an illicit recreational drug in combination with other drugs. To evaluate the potential of EAM-2201 as a perpetrator of drug–drug interactions, the inhibitory effects of EAM-2201 on major drug-metabolizing enzymes, cytochrome P450s (CYPs) and uridine 5′-diphospho-glucuronosyltransferases (UGTs) were evaluated in pooled human liver microsomes using liquid chromatography–tandem mass spectrometry (LC-MS/MS). EAM-2201 at doses up to 50 µM negligibly inhibited the activities of eight major human CYPs (1A2, 2A6, 2B6, 2C8, 2C9, 2C19, 2D6 and 3A4) and five UGTs (1A1, 1A4, 1A6, 1A9 and 2B7) in human liver microsomes. EAM-2201 exhibited time-dependent inhibition of CYP2C8-catalyzed amodiaquine *N*-deethylation, CYP2C9-catalyzed diclofenac 4′-hydroxylation, CYP2C19-catalyzed [*S*]-mephenytoin 4′-hydroxylation and CYP3A4-catalyzed midazolam 1′-hydroxylation with *K_i_* values of 0.54 µM (*k*_inact_: 0.0633 min^−1^), 3.0 µM (*k*_inact_: 0.0462 min^−1^), 3.8 µM (*k*_inact_: 0.0264 min^−1^) and 4.1 µM (*k*_inact_: 0.0250 min^−1^), respectively and competitively inhibited UGT1A3-catalyzed chenodeoxycholic acid 24-acyl-glucuronidation, with a *K_i_* value of 2.4 µM. Based on these in vitro results, we conclude that EAM-2201 has the potential to trigger in vivo pharmacokinetic drug interactions when co-administered with substrates of CYP2C8, CYP2C9, CYP2C19, CYP3A4 and UGT1A3.

## 1. Introduction

**Figure 1:**
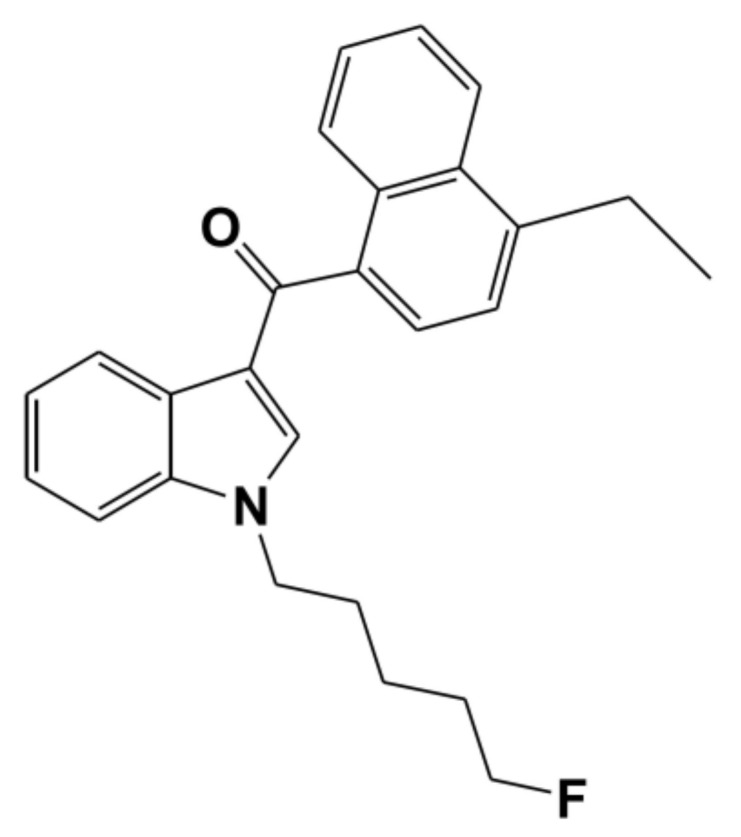
Chemical structure of EAM-2201.

EAM-2201, (4-ethyl-1-naphthalenyl)[1-(5-fluoropentyl)-1*H*-indol-3-yl]-methanone (Figure 1), is a synthetic cannabinoid receptor agonist that mimics the psychoactive effects of the principal psychoactive component of cannabis, ∆9-tetrahydrocannabinol (THC) [1,2,3,4,5]. EAM-2201 was identified for the first time in herbal products seized in 2012 and the use of synthetic cannabinoids, including EAM-2201, is increasingly prevalent [3,4,6,7]. EAM-2201 has been detected in forensic samples obtained in suspected impaired driving cases and in a postmortem case [8,9]. EAM-2201 is extensively metabolized to 37 metabolites in human liver microsomes and human cDNA-overexpressed cytochrome P450 (CYP) enzymes including CYPs 1A2, 2B6, 2C8, 2C9, 2C19, 2D6, 2J2, 3A4 and 3A5 via mono- and di-hydroxylation at the naphthalene moiety, indole moiety and pentyl chain; dehydrogenation at ethyl groups; oxidative defluorination at the fluoropentyl chain; dihydrodiol formation; *N*-dealkylation; and carboxylation [10].

Drug abusers frequently take drugs of abuse in combination with various therapeutic drugs; therefore, it is necessary to evaluate whether abused drugs are victims or perpetrators in drug-metabolizing enzyme mediated drug-drug interactions. CYP and uridine-5′- diphospho-glucuronosyltransferases (UGT) enzymes play major roles in drug metabolism [11,12,13]. The inhibitory effects of chemical derivatives of EAM-2201, such as AM-2201 [14] and MAM-2201 [15] and phytocannabinoids, such as THC, cannabidiol and cannabinol [16,17,18,19,20,21,22,23,24,25], on major human CYPs and UGT enzyme activities have been evaluated using human liver microsomes or recombinant CYP and UGT enzymes. EAM-2201 was detected with other synthetic cannabinoids including AB-CHMINACA, AB-FUBINACA, AM-2201, 5F-OMB, 5F-APINACA, STS135, THJ 2201, JWH-122 and XLR-11 in plasma samples of a postmortem case and recreational users [8,9]. Because EAM-2201 has been used with other drugs in combination, it is necessary to evaluate the possibility of EAM-2201 as the perpetrator of drug-drug interactions. However, there have been no reports on the effects of EAM-2201 on drug metabolizing enzymes.

In this study, the inhibitory potential of EAM-2201 on eight major human CYPs (1A2, 2A6, 2B6, 2C8, 2C9, 2C19, 2D6 and 3A4) and six UGTs (1A1, 1A3, 1A4, 1A6, 1A9 and 2B7) was evaluated using pooled human liver microsomes to predict the possibility of EAM-2201-induced drug–drug interactions.

## 2. Results

**Figure 2:**
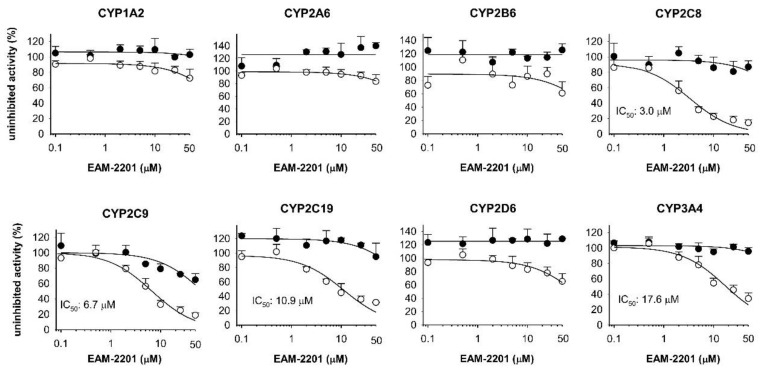
The inhibitory effects of EAM-2201 on CYP1A2-mediated phenacetin *O*-deethylase, CYP2A6-mediated coumarin 7-hydroxylase, CYP2B6-mediated bupropion hydroxylase, CYP2C8-catalyzed amodiaquine *N*-deethylase, CYP2C9-catalyzed diclofenac 4′-hydroxylase, CYP2C19-mediated [*S*]-mephenytoin 4′-hydroxylase, CYP2D6-mediated bufuralol 1′-hydroxylase and CYP3A4-mediated midazolam 1′-hydroxylase activities in ultrapooled human liver microsomes with (○) and without (●) 30-min preincubation in the presence of reduced β-nicotinamide adenine dinucleotide phosphate (NADPH) at 37 °C. The cocktail substrate concentrations used to assess IC_50_ values were as follows: 50 µM phenacetin, 2.5 µM coumarin, 2.0 µM amodiaquine, 10 µM diclofenac, 100 µM [*S*]-mephenytoin, 5.0 µM bufuralol and 2.5 µM midazolam. Inhibition of CYP2B6 was evaluated separately using 50 µM bupropion. The data are presented as the mean ± standard deviation (SD; *n* = 3).

EAM-2201 at a concentration of 50 µM negligibly inhibited CYP1A2-mediated phenacetin *O*-deethylation, CYP2A6-mediated coumarin 7-hydroxylation, CYP2B6-mediated bupropion hydroxylation, CYP2C8-catalyzed amodiaquine *N*-deethylation, CYP2C9-catalyzed diclofenac 4′-hydroxylation, CYP2C19-mediated [*S*]-mephenytoin 4′-hydroxylation, CYP2D6-mediated bufuralol 1′-hydroxylation and CYP3A4-mediated midazolam 1′-hydroxylation activities in human liver microsomes (Figure 2).

However, 30-min preincubation of human liver microsomes with EAM-2201 and reduced β-nicotinamide adenine dinucleotide phosphate (NADPH) resulted in strong inhibition of CYP2C8-catalyzed amodiaquine *N*-deethylation, CYP2C9-catalyzed diclofenac 4′-hydroxylation, CYP2C19-mediated [*S*]-mephenytoin 4′-hydroxylation and CYP3A4-mediated midazolam 1′-hydroxylation activities, with IC_50_ values of 3.0, 6.7, 10.9 and 17.6 µM, respectively (Figure 2), indicating that EAM-2201 is a potent time-dependent inhibitor of CYP2C8, CYP2C9, CYP2C19 and CYP3A4.

**Figure 3:**
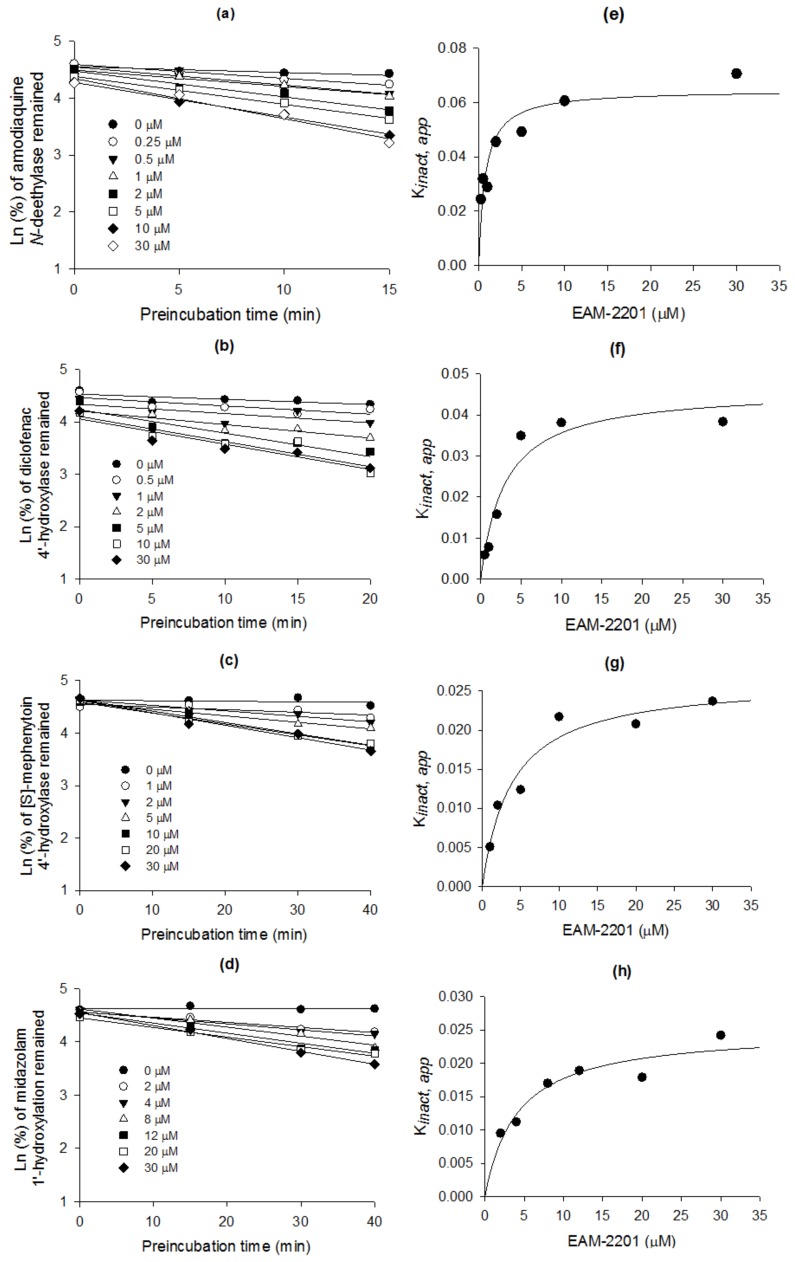
Inactivation kinetics of (**a**) *N*-desethylamodiaquine formation from amodiaquine; (**b**) 4′-hydroxydiclofenac formation from diclofenac; (**c**) 4′-hydroxy-[*S*]-mephenytoin from [*S*]-mephenytoin; and (**d**) 1′-hydroxymidazolam from midazolam in human microsomes by various concentrations of EAM-2201. Relationship between *k*_obs_ and EAM-2201 concentrations to estimate *k*_inact_ and *K_i_* values for (**e**) CYP2C8-mediated amodiaquine *N*-deethylation; (**f**) CYP2C9-mediated diclofenac 4′-hydroxylation; (**g**) CYP2C19-mediated [*S*]-mephenytoin 4′-hydroxylation; and (**h**) CYP3A4-mediated midazolam 1′-hydroxylation.

EAM-2201 decreased CYP2C8-mediated amodiaquine *N*-deethylation, CYP2C9-catalyzed diclofenac 4′-hydroxylation, CYP2C19-mediated [*S*]-mephenytoin 4′-hydroxylation and CYP3A4-mediated midazolam 1′-hydroxylation with increasing pre-incubation time, in a concentration-dependent manner (Figure 3a–d). The apparent *K_i_* and *k*_inact_ values were 0.54 µM and 0.0633 min^−1^, respectively, for CYP2C8-mediated amodiaquine *N*-deethylation, 3.0 µM and 0.0462 min^−1^ for CYP2C9-catalyzed diclofenac 4′-hydroxylation, 3.8 µM and 0.0264 min^−1^ for CYP2C19-mediated [*S*]-mephenytoin 4′-hydroxylation and 4.1 µM and 0.0250 min^−1^ for CYP3A4-mediated midazolam 1′-hydroxylation (Figure 3e–h, Table 1).

**Figure 4:**
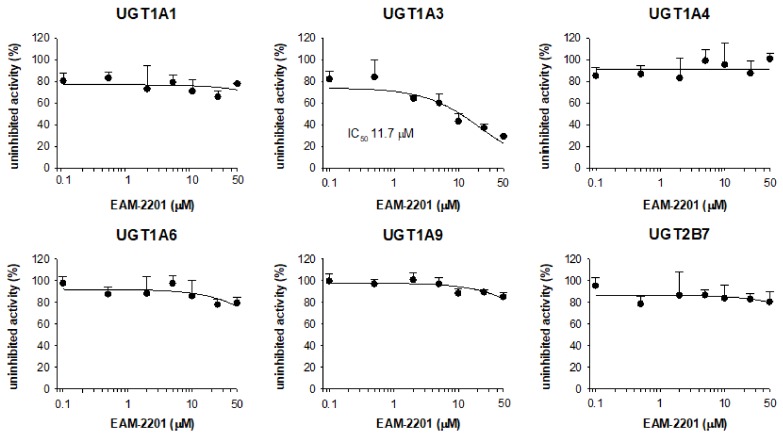
The inhibitory effects of EAM-2201 on the activities of six uridine 5′-diphospho-glucuronosyltransferases (UGT) enzymes in ultrapooled human liver microsomes. The cocktail UGT substrate concentrations were as follows: 0.5 µM SN-38 for UGT1A1, 2 µM chenodeoxycholic acid for UGT1A3, 0.5 µM trifluoperazine for UGT1A4, 1 µM *N*-acetylserotonin for UGT1A6, 0.2 µM mycophenolic acid for UGT1A9 and 1 µM naloxone for UGT2B7. The data are presented as the mean ± SD (*n* = 3).

**Figure 5:**
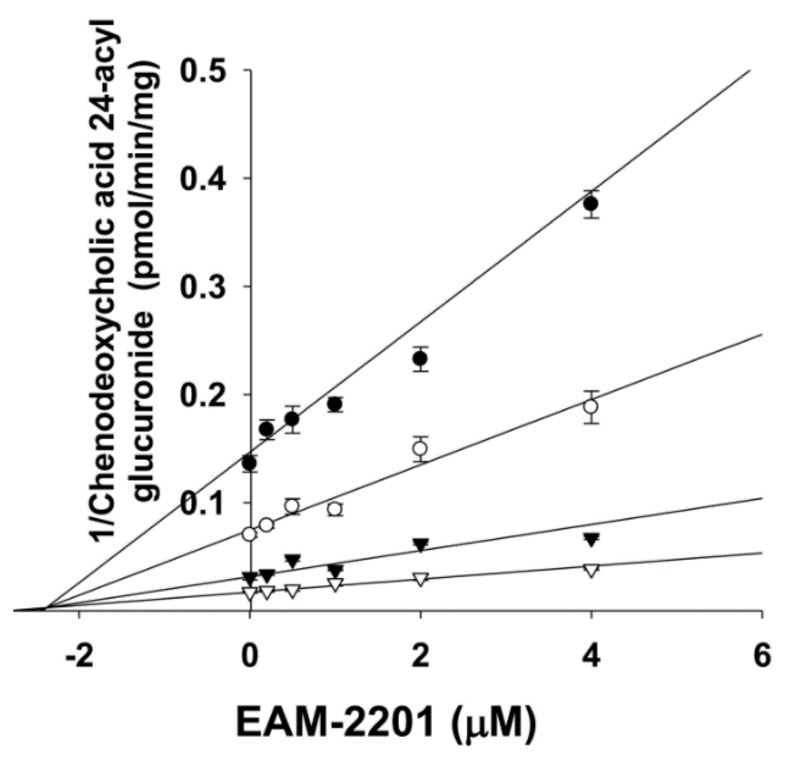
Representative Dixon plot showing the inhibitory effects of EAM-2201 on UGT1A3-catalyzed chenodeoxycholic acid 24-acylglucuronidation in human liver microsomes. Each symbol represents a particular concentration of chenodeoxycholic acid: ●, 1 µM; ○, 2 µM; ▼, 5 µM; and ▽, 10 µM. The data are presented as the mean ± SD (*n* = 3).

The inhibitory effects of EAM-2201 on six major human UGT enzymes were evaluated in human liver microsomes and EAM-2201 potently inhibited UGT1A3-catalyzed chenodeoxycholic acid 24-acyl-glucuronidation, with an IC_50_ value of 11.7 µM (Figure 4). EAM-2201 negligibly inhibited UGT1A1-catalyzed SN-38 glucuronidation, UGT1A4-catalyzed trifluoperazine *N*-glucuronidation, UGT1A6-catalyzed *N*-acetylserotonin glucuronidation, UGT1A9-catalyzed mycophenolic acid glucuronidation and UGT2B7-catalyzed naloxone 3-β-d-glucuronidation at 50 µM in human liver microsomes (Figure 4). EAM-2201 competitively inhibited UGT1A3-catalyzed chenodeoxycholic acid 24-acyl-glucuronidation with a *K_i_* value of 2.4 µM in human liver microsomes (Figure 5).

## 3. Discussion

We evaluated the inhibitory effects of EAM-2201 on CYP and UGT activities for the first time, using human liver microsomes. EAM-2201 negligibly inhibited the activities of eight major human CYPs but showed time-dependent inhibition of CYP2C8, CYP2C9, CYP2C19 and CYP3A4 enzyme activities in human liver microsomes (Figure 2). However, AM-2201 and MAM-2201, chemical derivatives of EAM-2201, potently inhibited CYP2C9 with *K_i_* values of 3.9 and 5.6 µM, respectively and CYP3A4 with *K_i_* values of 4.0 and 5.4 µM, respectively; and exhibited time-dependent inhibition of CYP2C8 activity in human liver microsomes [14,15]. The number of metabolites of EAM-2201 previously reported in human liver microsomes (37 metabolites) was greater than those of AM-2201 and MAM-2201 (19 metabolites) [10,26], suggesting that EAM-2201 is a better time-dependent inhibitor than AM-2201 and MAM-2201.

EAM-2201 showed potent time-dependent inhibition of CYP2C8-catalyzed amodiaquine *N*-deethylation, with a *K_i_* of 0.54 µM and *k*_inact_ of 0.0633 min^−1^ (Table 1) and its inactivation efficiency (*k*_inact_/*K_i_* = 117.2 mL/µmol/min) was higher than those of its chemical derivatives, such as AM-2201 (*k*_inact_/*K_i_* = 25.8 mL/µmol/min) [14] and MAM-2201 (*k*_inact_/*K_i_* = 73.8 mL/µmol/min) [15] and of typical CYP2C8 inhibitors such as phenelzine (*k*_inact_/*K_i_* = 3.13 mL/µmol/min) and amiodarone (*k*_inact_/*K_i_* = 0.57 mL/µmol/min) [27]. These results indicate that EAM-2201 may be a potent time-dependent inhibitor of CYP2C8 and may inhibit the metabolism of CYP2C8 substrate drugs, including cerivastatin, MAM-2201, paclitaxel, repaglinide and sorafenib [26,28].

EAM-2201 was a potent time-dependent inhibitor of CYP2C9-catalyzed diclofenac hydroxylation, with a *K_i_* value of 3.0 µM and *k*_inact_ of 0.0462 min^−1^ (Table 1), based on comparing the inactivation efficiency of EAM-2201 (*k*_inact_/*K_i_* = 15.4 mL/µmol/min) with those of typical CYP2C9 mechanism-based inhibitors such as tienilic acid (*k*_inact_/*K_i_* = 8.5 mL/µmol/min) and (±)-suprofen (*k*_inact_/*K_i_* = 1.1 mL/µmol/min) [29]. However, AM-2201 (*K_i_*, 3.9 µM), MAM-2201 (*K_i_*, 5.6 µM), THC (*K_i_*, 1.31 µM), cannabinol (*K_i_*, 1.29 µM) and cannabidiol (*K_i_*, 9.88 µM) inhibited mixed or competitively CYP2C9-catalyzed diclofenac hydroxylation in human liver microsomes [14,15,21]. EAM-2201 may cause drug interactions with CYP2C9 substrates such as celecoxib, diclofenac, glyburide, losartan, phenytoin, tolbutamide, torasemide, *S*-warfarin, AM-2201 and MAM-2201 [26,30,31].

EAM-2201 exhibited time-dependent inhibition of CYP2C19-mediated [*S*]-mephenytoin 4′-hydroxylation with a *K_i_* value of 3.8 µM and *k*_inact_ of 0.0264 min^−1^ (Table 1) and its inactivation efficiency of CYP2C19 (*k*_inact_/*K_i_* = 6.9 mL/µmol/min) was comparable or lower than those of typical mechanism-based CYP2C19 inhibitors, such as clopidogrel (*k*_inact_/*K_i_* = 3.9 mL/µmol/min) and ticlopidine (*k*_inact_/*K_i_* = 22.2 mL/µmol/min) [32,33]. However, AM-2201 and MAM-2201 did not inhibit CYP2C19-catalyzed [*S*]-mephenytoin hydroxylation in human liver microsomes [14,15] but cannabidiol (*K_i_*, 0.793 µM) and THC (*K_i_*, 1.93 µM) showed mixed-type inhibition of [*S*]-mephenytoin hydroxylation activity in human recombinant CYP2C19 enzymes [25]. EAM-2201 may cause drug interactions with CYP2C19 substrates such as diazepam, lansoprazole, omeprazole, rabeprazole, and voriconazole [34].

EAM-2201 exhibited time-dependent inhibition of CYP3A4-catalyzed midazolam 1′-hydroxylation with a *K_i_* value of 4.1 µM and *k*_inact_ of 0.0250 min^−1^ (Table 1) but AM-2201 (*K_i_*, 4.0 µM) and MAM-2201 (*K_i_*, 5.4 µM) showed competitive and noncompetitive inhibition of midazolam 1′-hydroxylation, respectively, in human liver microsomes [14,15]. Cannabidiol competitively inhibited CYP3A4-catalyzed diltiazem *N*-demethylation in human liver microsomes (*K_i_*, 6.14 µM) and human recombinant CYP3A4 enzymes (*K_i_*, 1.0 µM) but cannabinol and THC did not inhibit CYP3A4 activity [18]. The inactivation efficiency (*k*_inact_/*K_i_*) values of CYP3A4 time-dependent inhibitors with a reported clinical drug–drug interaction, such as clarithromycin, troleandomycin and verapamil were 2.8, 172.2 and 11.2 mL/µmol/min, respectively, in human liver microsomes [35]; but the *k*_inact_/*K_i_* value of EAM-2201 for CYP3A4 was 6.1 mL/µmol/min. Therefore, EAM-2201 can cause clinical drug–drug interactions with CYP3A4 substrates such as atorvastatin, cyclosporine, clarithromycin, estradiol, felodipine, lovastatin, MAM-2201, nifedipine, simvastatin and tacrolimus [36] via the time-dependent inhibition of CYP3A4.

EAM-2201 negligibly inhibited UGT1A1, UGT1A4, UGT1A6, UGT1A9 and UGT2B7 activities in human liver microsomes but showed potent competitive inhibition of UGT1A3-catalyzed chenodeoxycholic acid 24-acyl glucuronidation, with a *K_i_* value of 2.4 µM, similar to those of AM-2201 (*K_i_*, 4.3 µM), MAM-2201 (*K_i_*, 5.0 µM) and the selective UGT1A3 inhibitor glycyrrhetinic acid (IC_50_, 4.3 μM), in human liver microsomes [14,15]. Although cannabidiol and cannabinol inhibited UGT1A9 and UGT2B7 activity in human liver and intestinal microsomes [22], these results suggest that the concomitant abuse of EAM-2201 with UGT1A3 substrates, such as chenodeoxycholic acid, fimasartan, losartan, candesartan, zolarsartan, or JWH-018 [37,38,39,40] may possibly result in drug interactions.

There have been no direct reports on human EAM-2201 pharmacokinetics, which would be necessary for predicting EAM-2201-induced drug interaction potential. Serum EAM-2201 concentrations in plasma samples from recreational users and a postmortem case ranged from 0.26 to 4.1 nM [8,9] but these plasma concentrations do not reflect tissue concentrations, particularly liver concentrations. Our in vitro results suggest that EAM-2201 should be examined in terms of potential in vivo pharmacokinetic drug–drug interactions caused by time-dependent inhibition of CYP2C8, CYP2C9, CYP2C19 and CYP3A4 activities and competitive inhibition of UGT1A3 activity.

## 4. Materials and Methods

### 4.1. Chemicals and Reagents

EAM-2201 was obtained from Cayman Chemical Company (Ann Arbor, MI, USA). Acetaminophen, *N*-acetylserotonin, alamethicin, chenodeoxycholic acid, coumarin, diclofenac, dimethyl sulfoxide (DMSO), 7-hydroxycoumarin, midazolam, mycophenolic acid, naloxone, naloxone 3-β-d-glucuronide, NADPH, phenacetin, trifluoperazine, Trizma base and uridine 5′-diphosphoglucuronic acid (UDPGA) were purchased from Sigma-Aldrich (St. Louis, MO, USA). ^13^C_2_,^15^N-acetaminophen, bufuralol, *N*-desethylamodiaquine, 1′-hydroxybufuralol, d_9_-1-hydroxybufuralol, 4-hydroxydiclofenac, 4-hydroxymephenytoin, 1′-hydroxymidazolam, [*S*]-mephenytoin and ultrapooled human liver microsomes (150 donors) were purchased from Corning Life Sciences (Woburn, MA, USA). *N*-Acetylserotonin β-d-glucuronide, chenodeoxycholic acid 24-acyl-β-glucuronide, mycophenolic acid β-d-glucuronide and SN-38 glucuronide were obtained from Toronto Research Chemicals (Toronto, ON, Canada). SN-38 was obtained from Santa Cruz Biotechnology (Dallas, TX, USA). Acetonitrile, methanol and water were of liquid chromatography–mass spectrometry (LC-MS) grade and purchased from Fisher Scientific Co. (Fair Lawn, NJ, USA). All other chemicals were of the highest quality available.

### 4.2. Inhibitory Effects of EAM-2201 on Eight Major CYP Activities in Human Liver Microsomes

The inhibitory potential (IC_50_ values) of EAM-2201 on CYP1A2, CYP2A6, CYP2C8, CYP2C9, CYP2C19, CYP2D6 and CYP3A4 were evaluated using ultrapooled human liver microsomes and a cocktail of seven CYP substrates followed by LC-tandem mass spectrometry (LC-MS/MS) as previously described [41]. Each incubation mixture was prepared in a total volume of 100 µL as follows: pooled human liver microsomes (5.2 pmol CYP), 1.0 mM NADPH, 10 mM magnesium chloride, 50 mM potassium phosphate buffer (pH 7.4), various concentrations of EAM-2201 in DMSO (final concentrations of 0.1–50 µM, 0.5% DMSO) and a cocktail of seven CYP probe substrates in 50% acetonitrile (0.5% acetonitrile), including 50 µM phenacetin, 2.5 µM coumarin, 2.0 µM amodiaquine, 10 µM diclofenac, 100 µM [*S*]-mephenytoin, 5 µM bufuralol and 2.5 µM midazolam in 50% acetonitrile. After a 3-min preincubation at 37 °C, the reactions were initiated by adding NADPH and incubation proceeded for 15 min at 37 °C in a shaking water bath. The reactions were stopped by adding 100 µL of ice-cold methanol containing internal standards (^13^C_2_,^15^N-acetaminophen for acetaminophen and *N*-desethylamodiaquine; d_9_-1′-hydroxybufuralol for 4′-hydroxydiclofenac, 7-hydroxycoumarin, 4′-hydroxymephenytoin, 1′-hydroxybufuralol and 1′-hydroxy-midazolam). The incubation mixtures were centrifuged at 13,000× *g* for 4 min at 4 °C and 50 µL of each supernatant was diluted in 50 µL of water. Aliquots (5 µL) of the diluted supernatants were analyzed using LC-MS/MS. All assays were performed in triplicate and the mean values were used in calculations. To measure the time-dependent inhibition of seven CYP activities, various concentrations of EAM-2201 (0.1–50 µM) were pre-incubated for 30 min with human liver microsomes in the presence of NADPH. Each reaction was initiated by adding the seven-CYP probe substrate cocktail described above.

The inhibitory effects (IC_50_ values) of EAM-2201 on CYP2B6-catalyzed bupropion 4-hydroxylase activity were determined in ultrapooled human liver microsomes using LC-MS/MS [41]. Each incubation mixture was prepared in a total volume of 100 µL as follows: pooled human liver microsomes (5.2 pmol CYP), 1.0 mM NADPH, 10 mM magnesium chloride, 50 mM potassium phosphate buffer (pH 7.4), various concentrations of EAM-2201 in DMSO (final 0.1–50 µM, 0.5% DMSO) and CYP2B6-selective substrate and 50 µM bupropion. After a 3-min preincubation at 37 °C, reactions were initiated by adding NADPH, followed by incubation for 15 min at 37 °C in a shaking water bath. The reactions were stopped by adding 100 µL of ice-cold methanol containing d_9_-1-hydroxybufuralol (internal standard). The incubation mixtures were centrifuged at 13,000× *g* for 4 min at 4 °C and 50 µL of each supernatant was diluted in 50 µL of water. Aliquots (5 µL) of the diluted supernatants were analyzed by LC-MS/MS. All incubations were performed in triplicate and average values were used in calculations. To evaluate time-dependent inhibition of CYP2B6 activity, various concentrations of EAM-2201 (final 0.1–50 µM) were pre-incubated for 30 min with human liver microsomes in the presence of NADPH. Each reaction was commenced by adding 50 µM bupropion as described above.

### 4.3. Inhibitory Effects of EAM-2201 on Six Major UGTs in Human Liver Microsomes

The inhibitory effects of EAM-2201 on UGT1A1, UGT1A3, UGT1A4, UGT1A6, UGT1A9 and UGT2B7 were evaluated using LC-MS/MS and the cocktail of UGT substrates as previously described [41]. Each incubation mixture was prepared in a final volume of 100 µL as follows: ultrapooled human liver microsomes (0.2 mg/mL), 5 mM UDPGA, 10 mM magnesium chloride, alamethicin (25 µg/mL), 50 mM Tris buffer (pH 7.4), various concentrations of EAM-2201 in DMSO (final concentrations of 1–50 µM, 0.5% DMSO) and the UGT enzyme-specific substrates of two cocktail sets (set A: 0.5 µM SN-38, 2 µM chenodeoxycholic acid and 0.5 µM trifluoperazine; set B: 1 µM *N*-acetylserotonin, 0.2 µM mycophenolic acid and 1 µM naloxone, acetonitrile content, 0.5%). After a 5-min preincubation at 37 °C, the reactions were initiated by adding UDPGA and incubated for 60 min at 37 °C in a shaking water bath. Reactions were terminated by adding 50 µL of ice-cold acetonitrile containing internal standards (propofol glucuronide for chenodeoxycholic acid 24-acyl-β-glucuronide and mycophenolic acid glucuronide and meloxicam for SN-38 glucuronide, trifluoperazine glucuronide, *N*-acetylserotonin β-d-glucuronide and naloxone 3-β-d-glucuronide). The incubation mixtures were centrifuged at 13,000× *g* for 4 min at 4 °C. Supernatant from sets A and B (50 µL of each) was mixed and aliquots (5 µL) were analyzed by LC-MS/MS. All assays were performed in triplicate and the mean values were used in calculations.

### 4.4. Time-Dependent Inhibition of CYP2C8, CYP2C9, CYP2C19 and CYP3A4 Activities by EAM-2201

The time-dependent inhibitory effects of EAM-2201 on CYP2C8, CYP2C9, CYP2C19 and CYP3A4 activities in ultrapooled human liver microsomes were evaluated using time- and concentration-dependent inhibition assays. Ultrapooled human liver microsomes (26.0 pmol CYP) were pre-incubated with various concentrations of EAM-2201 in DMSO (0.5% DMSO) in 50 mM potassium phosphate buffer (pH 7.4) in the presence of NADPH for predetermined time periods: 5–15 min for CYP2C8 activity, 5–20 min for CYP2C9 activity and 15–40 min for CYP2C19 and CYP3A4 activities. Aliquots (10 µL) of the preincubated mixtures were withdrawn at predetermined times after incubation and added to other tubes containing specific CYP substrates (2 µM amodiaquine for CYP2C8, 10 µM diclofenac for CYP2C9, 100 µM [*S*]-mephenytoin for CYP2C19, or 2 µM midazolam for CYP3A4 in 50% acetonitrile), 1 mM NADPH, 50 mM potassium phosphate buffer (pH 7.4) and 10 mM magnesium chloride in 90-µL reaction mixtures. The second reactions were terminated after 10 min by adding 100 µL of ice-cold methanol containing d_9_-1′-hydroxybufuralol (internal standard). The incubation mixtures were centrifuged at 13,000× *g* for 4 min at 4 °C and 50 µL of each supernatant was diluted in 50 µL of water. Aliquots (5 µL) of the diluted supernatants were analyzed using LC-MS/MS.

### 4.5. Enzyme Kinetic Analysis

To determine the *K_i_* value of EAM-2201 on UGT1A3 inhibition, various concentrations of chenodeoxycholic acid (1–10 µM) and EAM-2201 in DMSO (0.5–8 µM, 0.5% DMSO) were incubated with human liver microsomes (0.15 mg/mL), 5 mM UDPGA, 10 mM magnesium chloride, alamethicin (25 µg/mL) and 50 mM Tris buffer (pH 7.4) in a total incubation volume of 100 µL. Reactions were initiated by adding UDPGA at 37 °C and stopped after 60 min by adding 100 µL ice-cold methanol containing propofol glucuronide (internal standard). The incubation mixtures were centrifuged (13,000× *g*, 4 min, 4 °C) and 50 µL of the supernatant was diluted in 50 µL of water. Aliquots (5 µL) of the diluted supernatants were analyzed using LC-MS/MS.

### 4.6. LC-MS/MS Analysis

The metabolites formed from CYP substrates were simultaneously quantified using our previously described LC-MS/MS method [41]. A tandem mass spectrometer (TSQ Quantum Access, Thermo Scientific, San Jose, CA, USA), equipped with an electrospray ionization (ESI) source coupled to a Nanospace SI-2 LC system (Tokyo, Japan) was used. The column and autosampler temperatures were 50 °C and 6 °C, respectively. Separations were performed on an Atlantis dC18 column (3 μm, 2.1 mm i.d. × 100 mm; Waters Corporation, Milford, MA, USA) using the gradient elution of a mixture of 5% methanol in 0.1% formic acid (*v*/*v*) (mobile phase A) and 95% methanol in 0.1% formic acid (*v*/*v*) (mobile phase B) at a flow rate of 0.25 mL/min: 10% mobile phase B for 2 min and 10% to 95% mobile phase B for 4 min. The ESI source settings in the positive ion mode were as follows: capillary voltage, 4200 V; vaporizer temperature, 350 °C; capillary temperature, 330 °C; sheath gas pressure, 35 psi; and auxiliary gas pressure, 15 psi. Quantification was performed by selected reaction monitoring (SRM) of the [M + H]^+^ ion and the related product ion for each metabolite: acetaminophen, 152.1 > 110.3; *N*-desethylamodiaquine, 328.1 > 283.0; 7-hydroxycoumarin, 163.0 > 107.2; 4-hydroxybupropion, 256.1 > 238.0; 4′-hydroxydiclofenac, 312.0 > 231.1; 4′-hydroxy-mephenytoin, 235.1 > 150.1; 1′-hydroxybufuralol, 278.1 > 186.1; 1′-hydroxymidazolam, 341.9 > 324.0; ^13^C_2_,^15^N-acetaminophen, 155.1 > 111.2; and d_9_-1′-hydroxybufuralol, 287.2 > 187.0. Analytical data were processed using Xcalibur software (Thermo Scientific).

The metabolites formed from the six UGT cocktail substrates were simultaneously measured using LC-MS/MS [41]. Metabolites were separated on an Atlantis dC18 column (3 μm, 2.1 mm i.d. × 100 mm; Waters Corporation) via gradient elution using a mixture of 5% acetonitrile in 0.1% formic acid (mobile phase A) and 95% acetonitrile in 0.1% formic acid (mobile phase B) at a flow rate of 0.2 mL/min: 100% mobile phase A for 1.7 min, 0 to 98% mobile phase B for 0.1 min and 98% mobile phase B for 3.2 min. The ESI source settings in both the positive and negative ion modes were as follows: capillary voltage, 4200 V; vaporizer temperature, 350 °C; capillary temperature, 330 °C; sheath gas pressure, 35 psi; and auxiliary gas pressure, 15 psi. Each metabolite was quantified via SRM in the negative ion mode: chenodeoxycholic acid 24-acyl-β-glucuronide, 567.2 > 391.0; mycophenolic acid glucuronide, 495.2 > 318.9; and propofol glucuronide (IS), 353.3 > 177.1; and in the positive ion mode: SN-38 glucuronide, 569.0 > 393.0; trifluoperazine glucuronide, 584.2 > 408.1; *N*-acetylserotonin-β-d-glucuronide, 395.2 > 219.0; naloxone 3-β-d-glucuronide, 504.0 > 310.0; and meloxicam (IS), 352.0 > 115.1. The data were processed with the aid of Xcalibur software.

### 4.7. Data Analysis

IC_50_ values (the concentration of the inhibitor associated with 50% inhibition of the original enzyme activity) were calculated by nonlinear regression analysis with Sigma Plot 12.0 software (Systat Software Inc., San Jose, CA, USA).

To determine the reversible inhibition constant (*K_i_*) and inhibition mode for UGT1A3, data obtained from enzyme kinetic inhibition were fitted to different built-in equations for competitive, noncompetitive, uncompetitive and mixed inhibition models using Enzyme Kinetics ver. 1.1 software (Systat Software Inc.), which automatically estimates the initial parameters for the selected models and uses the Levenberg–Marquardt algorithm to determine the parameter values. The best model was determined using Akaike’s information criterion as a measure of goodness of fit. The inhibition mode was verified by visual inspection of Lineweaver–Burk plots of enzyme kinetic data provided by Enzyme Kinetics software.

For time-dependent inhibition, the observed rates of CYP2C8, CYP2C9, CYP2C19 and CYP3A4 inactivation (*k*_obs_) at different EAM-2201 concentrations were calculated from the negative slopes of the lines using linear regression analysis of the natural logarithm of the remaining activity as a function of time. Then, the inhibitor concentration that supports half the maximal rate of inhibition (*K_i_*) and maximal rate of enzyme inhibition (*k*_inact_) values were calculated using the following equation with Enzyme Kinetics software:

*k*_obs_ = *k*_inact_ × *I*/(*K_i_* + *I*),

where *I* is the initial concentration of EAM-2201.

## 5. Conclusions

EAM-2201 was a potent time-dependent inhibitor of CYP2C8-catalyzed amodiaquine *N*-deethylation, CYP2C9-catalyzed diclofenac 4′-hydroxylation, CYP2C19-catalyzed [*S*]-mephenytoin 4′-hydroxylation and CYP3A4-catalyzed midazolam 1′-hydroxylation, with *K_i_* values of 0.54, 3.0, 3.8 and 4.1 µM, respectively and *k*_inact_ values of 0.0633, 0.0462, 0.0264 and 0.0250 min^−1^, respectively; and it competitively inhibited UGT1A3-catalyzed chenodeoxycholic acid 24-acyl-glucuronidation with a *K_i_* value of 2.4 µM in human liver microsomes. The possibility of in vivo pharmacokinetic drug interactions with EAM-2201 should be evaluated, owing to its inhibition of CYP2C8, CYP2C9, CYP2C19, CYP3A4 and UGT1A3 activities.

## Figures and Tables

**Table 1:** *K_i_* and *k*_inact_ values for the time-dependent inhibition of EAM-2201 on four cytochrome P450 (CYP) metabolic activities in ultrapooled human liver microsomes.

Enzyme Activity	CYP	*K_i_* (µM)	*k*_inact_ (min^−1^)
Amodiaquine *N*-deethylase	2C8	0.54	0.0633
Diclofenac 4′-hydroxylase	2C9	3.0	0.0462
[*S*]-Mephenytoin 4′-hydroxylase	2C19	3.8	0.0264
Midazolam 1′-hydroxylase	3A4	4.1	0.0250
